# Molecular mechanism of stellate cell activation and therapeutic strategy for liver fibrosis

**DOI:** 10.1186/1476-5926-2-S1-S3

**Published:** 2004-01-14

**Authors:** Norifumi Kawada

**Affiliations:** 1 Department of Hepatology, Graduate School of Medicine, Osaka City University, Osaka 545-8585, Japan

## Introduction

Hepatic stellate cells, which reside in the space of Disse in close contact with both sinusoidal endothelial cells and hepatocytes, play multiple roles in the pathophysiology of the liver [1]. Quiescent stellate cells represent a principal retinol-storing phenotype and metabolize a small amount of basement membrane-forming substrata such as laminine and type IV collagen. When liver injury occurs, they undergo transformation into myofibroblasts eliciting active proliferation in response to platelet-derived growth factor-BB (PDGF-BB) and insulin-like growth factor-1 (IGF-1), increased extracellular matrix (ECM) production, increased contractility that is accompanied by the expression of smooth muscle –-actin and the production of endothelin-1 (ET-1), secretion of transforming growth factor-beta (TGF-beta) and monocyte chemotactic protein-1 (MCP-1), retinoid loss, and exhibiting active apoptosis. Stellate cell activation is initiated by oxidative stress of lipid hydroperoxide and reactive aldehyde generated and released by damaged hepatocytes, by paracrine stimulation of PDGF-BB, IGF-1 and TGF-beta derived from activated Kupffer cells, endothelial cells, platelets and infiltrating leukocytes, and by early ECM changes including the production of a splice variant of cellular fibronectin (EIIIA isoform) [2-5]. Transcriptional activation by a zinc finger gene KLF6, which is induced at the very early stage of liver injury, AP-1 and CCAAT/enhancer binding protein (C/EBP) enhances gene expression regulating ECM accumulation [6]. Activated stellate cells are highly responsive to PDGF-BB and IGF-1 through the induction of receptors for individual growth factors, resulting in the activation of intracellular signal cascade including phosphorylation of tyrosine residues in each growth factor receptor molecule, mitogen activated protein kinase (MAPK), phosphatidil inositol 3-kinase (PI3-K), leading to DNA synthesis and proliferation [7,8]. TGF-beta is a key regulatory molecule for ECM metabolism and functions as an autocrine and a paracrine mediator. The impact of TGF-beta1 on liver fibrosis has been well documented in a TGF-beta1 knockout mouse model [9], in the remarkable attenuation of the development of liver fibrosis by using soluble type II TGF-beta receptor [10], and in adenoviral delivery of dominant-negative TGF-beta receptor [11]. Role of Smad cascade in TGF-beta signaling has been well characterized. Increased contractility after activation, in particular induced by ET-1, causes constriction of sinusoids, leading to a persistent disturbance of intrahepatic microcirculation and portal hypertension [12,13]. Thus, analysis of the molecular mechanism underlying stellate cell activation is assumed to be essential for the development of effective therapy against liver fibrosis.

## Proteomics

The genome project will finish by 2003 and will reveal the sequence of all genes encoded in the genome for a total of 3,000 Mb [14,15]. This urges to develop genomics research permitting the analysis of more than 10,000 genes at one time by using gene chip technology. Such a newly developing approach has accelerated the discovery of genetic or common disease genes. Although calculated number of genes encoded in the genome is though to be about 40,000, 5,000 – 6,000 proteins are estimated to be generated in each type of cell and hence only a part of the genes in the genome are expressed in a cell- and tissue-specific manner. Therefore, analysis of cellular proteins, especially the change in their expression level and their post-transcriptional modification, is definitely required.

The proteome (**prote**in + gen**ome**), or proteomics, refers to the total protein profile of a given cell or tissue type. Advantage of proteomics is referred to more accurate picture of the genome's plan by measuring proteins directly because i) there exists difference between the rates of degradation of individual mRNAs and proteins and ii) proteins are received post-translational modification, e.g. phosphorylation and glycation. Disadvantage of proteomics may be i) requirement of "skilled staff" for 2-dimensional PAGE, ii) low sensitivity, iii) failure of the detection of some of the most interesting proteins, such as membrane receptor and low-abundance signal molecules, and iv) unavailability of amplification method for proteins like PCR. Recent advancement of technology related to proteomics will overcome these disadvantages in the near future.

Proteomics involves the following experimental steps. First, proteins are separated by two-dimensional polyacrylamide gel electrophoresis (2-D PAGE) [16]. This technique combines isoelectric focusing (IEF) in the first dimension with sodium dodecyl sulfate (SDS) PAGE in the second dimension, and it is capable of separating several thousands of proteins on a single 2-D gel. Second, individual proteins are identified and characterized by mass spectrometric techniques such as matrix-assisted laser desorption ionization (MALDI) or electrospray ionization (ESI) mass spectrometry [17,18]. Advances in these techniques of protein analysis using mass spectrometry (MS) has made it possible to increase the sensitivity of analysis (less than 100 fmol of proteins can be analyzed) and shorten the time for analysis (only 30 min is required for the partial sequencing of one protein spot). Moreover, the tandem mass (MS/MS) method directly represents the amino acid sequence of the protein spot analyzed without any pre-purification by such methods as high-performance liquid chromatography, resulting in the determination of the protein by reference to the database.

In practice, samples were prepared from quiescent or activated stellate cells, intact or fibrotic liver, and conditioned medium. They were dissolved in a lysis buffer composed of 7 M urea, 2 M thiourea, 4% (w/v) CHAPS, 2% (v/v) ampholine, and 1% DTT and applied overnight to Immobilone DryStrip (pH 4–7, Pharmacia). After IEF, two-dimensional SDS-PAGE was performed in 9–18% acrylamide gradient gels. Proteins were visualized by silver staining. The protein spots of interest were excised from the 2-D gels and digested overnight at 37–C in a buffer containing trypsin. After a brief purification, they were applied to Q-TOF mass spectrometer. The MS mode was used to scan samples for detectable peptides. Subsequently, the MS/MS mode was used to fragment individual peptides, and from the resulting MS/MS spectra the amino acid sequence was deduced for each peptide. Finally, the proteins were identified after searching the obtained amino acid sequences against the SwissProt and GenBank database.

A total of 308 protein spots from stellate cells have been successfully identified. These include 225 protein spots derived from stellate cell lysate and 83 spots of secreted proteins. Among the identified proteins were Cu^2+^/Zn^2+ ^superoxide dismutase, plasminogen activator inhibitor 1, ubiquitin, vimentin, and several heat-shock proteins. Several proteins whose expression levels were up- or down-regulated in the course of stellate cell activation were identified. These include actin-binding proteins, such as calcyclin, calgizzarin and F-actin capping protein, proteases, such as cathepsin D, gamma enolase, and serine protease, and neural cell adhesion molecule (N-CAM). Interesting was that we detected the difference in protein level between "*in vitro*" activated stellate cells and "*in vivo*" activated stellate cells. For instance, FK506 binding protein 6, heat shock protein 90-beta and oestronectin were detected in "*in vitro*", but not "*in vivo*" activated stellate cells. In addition, protein level of coffilin, destrin and proteasome delta chain was much higher in "*in vitro*" activated stellate cells than in "*in vivo*" ones [19]. Thus, we should keep in mind that the culture model does not always precisely reflect the cellular metabolism at the physiological situation.

## Discovery of STAP

In the course of the proteomics approach, we successfully isolated a novel protein dabbed STAP after stellate cell activation-associated protein and cloned its cDNA (*stap*) [20]. Rat STAP protein with pI 6 and molecular mass of 21 kDa was found to be present in stellate cells at their quiescent stage and to be markedly up-regulated during the course of their activation in both *in vivo *and in culture. Induction of STAP protein coincided with the expression of another activation markers of the cells, such as PDGFR-beta, smooth muscle –-actin and N-CAM. STAP expression was dramatically augmented in a fibrotic liver model of rats induced by thioacetamide, indicating a profound role of STAP in the rat hepatic pathophysiology including fibrosis. Further information obtained by immunohistochemistry of whole body organs of rats revealed that STAP would be a promising marker for vitamin A-storing cells in the extra-hepatic organs; STAP was detected in pancreatic stellate cells and renal mesangial cells as well as mesenchymal cells in the submucosal layer of the stomach and the intestines where the presence of vitamin A-storing cells was confirmed previously.

Homologous sequences to rat *stap *cDNA were found in human genome DNA database. By using the data, we designed primers and performed RT-PCR to amplify human *stap *cDNA by using cDNA derived from mRNA of human cultured stellate cells as a template. As a result, a 658 bp cDNA was obtained. The deduced amino acid sequence of the cDNA had 94 and 97% identity to rat *stap *and unknown mouse full-length cDNA (accession number AK019410), respectively. Thus, we concluded this clone as human *stap*. Two histidine residues were found in human STAP as in rat STAP at 81 and 113 from the N-terminus that might be expected to be involved in heme binding sites from the similarity with amino acid sequences of hemoglobin and myoglobin. BLAST search identified that human *stap *locates on chromosome 17 between sialyltransferase and hypothetical protein FLJ22341 and is composed of 4 exons, spanning 111140 to 102176 in RP11-666A8 clone [21].

STAP mRNA was expressed in human various organs and Hep G2 cells. Immunohistochemistry revealed that STAP-positive cells in the liver were stellate cells. STAP expression was not augmented around inflammatory necrotic areas where myofibroblasts were accumulated. As rat STAP, recombinant human STAP exhibited the characteristics of a heme protein with the activity of myeloperoxidase and lipid hydroperoxide dehydrogenase, but not glutathione peroxidase. These results indicate that human STAP is a novel human heme protein with peroxidase activity and can be used as a marker for stellate cells in human liver tissues.

Very recently, another laboratories reported homologous cDNAs to STAP from mouse and human and named them as cytoglobin and histoglobin, respectively. In accordance with our results, both cytoglobin and histoglobin are hemoprotein and are expressed in multiple organs. Taken together, STAP consists of a new family of globin other than hemoglobin and myoglobin.

Overexpression of STAP in NIH 3T3 cells revealed that STAP may regulate cell growth via reducing p21 expression. This experiment further showed that STAP may up-regulate mRNA expression of type I collagen and TGF-beta, indicating a profound role of STAP in fibrotic gene expression.

Although molecular function of STAP is beginning to be uncovered, the data obtained to date indicate the profound role of STAP in fibrotic potential and vitamin A metabolism of stellate cells in the liver as well as in the extra-hepatic organs.

Thus, the proteomics approach is highly useful to analyze protein/gene expression of stellate cells in response to their activation in *in vitro *and *in vivo*. Such a global catalogue of protein expression will greatly increase understanding of stellate cell biology and the molecular mechanism of liver fibrosis.

## Therapeutic Strategy for Preventing Liver Fibrosis

Increasing numbers of studies have shown a variety of therapeutic approaches for liver fibrosis based on the molecular inhibition of stellate cell activation.

Among them, interferon (IFN) has a clinical potential to treat liver fibrosis by eliminating HCV virus from patients [22]. IFN-Gamma inhibits stellate cell activation, collagen transcription through directly acting onto IFN-responsive element, a proximal element within the human alpha 2(I) collagen (COL1A2) promoter, and TGF-beta/Smad signaling through STAT pathway [23,24].

In animal models, liver fibrosis could be inhibited by a soluble receptor consisting of a chimeric IgG at the extracellular portion of the TGF-beta type II receptor and by the adenovirus-mediated local expression of a dominant-negative type II TGF-beta receptor (AdCATb-TR) [10,11].

We reported that N-acetyl-L-cysteine triggers the degradation of PDGF receptor beta mediated by cathepsin B. N-acetyl-L-cysteine inhibits PDGF signaling, PDGF-dependent DNA synthesis, and in addition, affected the expression of TGF-beta receptor type II. N-acetyl-L-cysteine dramatically attenuated liver fibrosis in rat models [25].

HGF gene therapy is promising for the treatment of liver fibrosis. Ueki et al. reported that repeated transfections of the human HGF gene into skeletal muscle suppressed the increase of TGF-beta 1, inhibited fibrogenesis and hepatocyte apoptosis, and resolved completely fibrosis in the cirrhotic liver [26].

Thiazolidinediones are clinically used as antidiabetic agents, which are ligands for peroxisome proliferator-activated receptor gamma (PPARgamma). Thiazolidinediones and the other PPARgamma ligands, such as 15-J-PGJ2, have been reported to inhibit proliferation and smooth muscle –-actin expression in cultured stellate cells [27-29]. A more recent report demonstrated that administration of thiazolidinediones reduced ECM deposition and stellate cell activation in both toxic and cholestatic model of liver fibrosis in rats [30].
